# Serum 25(OH)D reflects clinical characterization in dogs with chronic enteropathies

**DOI:** 10.3389/fvets.2025.1677939

**Published:** 2025-10-17

**Authors:** Carla Giuditta Vecchiato, Anisa Bardhi, Antonio Maria Tardo, Lorenzo Foietta, Giacomo Biagi, Maria Chiara Sabetti, Federico Fracassi, Andrea Barbarossa, Marco Pietra

**Affiliations:** ^1^Department of Veterinary Medical Sciences, University of Bologna, Bologna, Italy; ^2^Department of Veterinary Sciences, University of Parma, Parma, Italy

**Keywords:** 25(OH)D, vitamin D, hypovitaminosis D, chronic enteropathy, protein losing enteropathy

## Abstract

**Introduction:**

In canine chronic enteropathies (CE) and protein-losing enteropathy (PLE), vitamin D deficiency is recognized as a negative prognostic factor, while 25(OH)D status in relation to other clinical phenotypes has been poorly investigated.

**Methods:**

This study aimed to describe differences in CE dogs according to their response to treatment and to reveal potential associations with retrospective clinical and diagnostic data.

**Results:**

A total of 91 dogs were obtained from clinical records and categorized based on their response to treatment into the following groups: food-responsive enteropathy (FRE, *n* = 39), microbiota-related modulation-responsive enteropathies (MrMRE, *n* = 26), immunosuppressant-responsive enteropathy (IRE, *n* = 16), and PLE (*n* = 10). 25(OH)D, determined by UHPLC–MS/MS from stored serum samples, differed significantly among groups (*p* < 0.001). Median levels were markedly lower in the PLE group (10.3 ng/mL; range 10–27) than in FRE (33 ng/mL; 10–68) and MrMRE (37 ng/mL; 10–61) groups (*p* < 0.001). IRE group (20 ng/mL; 10–43) also showed reduced concentrations relative to FRE and MrMRE (*p* = 0.006). A multivariable linear regression model obtained using data from 75/91 dogs, revealed that fructosamine and phosphorus were positively associated with 25(OH)D status, while increased c-reactive protein was associated with a lower 25(OH)D.

**Conclusion:**

In CE dogs, 25(OH)D is negatively affected by inflammation and reflects the severity of clinical characteristics and serum protein-related biomarkers.

## Introduction

Chronic enteropathies (CE) comprise a heterogeneous group of frequently encountered gastrointestinal disorders in dogs. These conditions are characterized by persistent or recurrent gastrointestinal signs, most commonly including diarrhea, vomiting, loss of appetite, and weight loss (1). CE encompass various clinical phenotypes, which are traditionally categorized based on the dog's response to a stepwise therapeutic approach aimed at achieving clinical remission (2). Cases complicated by protein-losing enteropathy (PLE) are considered the most severe and are associated with a poorer prognosis (3). Low vitamin D is common in dogs with chronic intestinal disorders. It is associated with increased disease severity in CE and PLE (4–6), higher inflammatory markers in CE (7), worse histopathological scores (8) and poorer outcomes both in CE and PLE (9, 10).

The development of hypovitaminosis D in CE is likely multifactorial, involving several interrelated pathways that have not yet been thoroughly investigated in dogs. Impaired absorption of fat and fat-soluble vitamins due to intestinal inflammation and mucosal damage is a major contributor (5, 8), supported by reduced serum cholesterol and α-tocopherol in affected dogs (4, 8). Systemic and gastrointestinal inflammation may further impair vitamin D status (7). Additionally, hypoalbuminemia can decrease vitamin D transport capacity, although direct loss of vitamin D-binding protein seems less relevant (8). Secondary disturbances such as hyperparathyroidism and hypocalcemia, along with potential effects of inflammatory cytokines and altered vitamin D receptor expression, may also contribute, whereas reduced dietary intake has not been consistently demonstrated as a primary cause (5, 6, 8).

Despite the recognized importance of vitamin D in CE, its measurement is not routinely performed.

While its correlation with the canine chronic enteropathy clinical activity index score (CCECAI) has been described (8), a potential association between vitamin D status and clinical phenotypes based on treatment response has not yet been reported.

This study hypothesized that, in line with the predictive potential of vitamin D, differences in vitamin D concentration would be associated with variations in the severity of clinical signs and response to treatment. To test this theory, a retrospective study was conducted on a population of dogs diagnosed with CE and with documented treatment responses, in which serum 25-hydroxyvitamin D - 25(OH)D- concentrations were measured. For these dogs, laboratory test results available in the patient database were also evaluated in order to investigate possible associations between vitamin D levels and other blood parameters.

## Materials and methods

In this study, adult dogs diagnosed with CE, that underwent the first clinical examination and blood sampling at the Veterinary Teaching Hospital of the University of Bologna between May 2021, and April 2023, were retrospectively included. Data obtained from medical records included signalment, history, comprehensive diagnostic evaluation, clinical and clinicopathological findings and follow-up examinations re-evaluated over time. For the purposes of the study, the following data were extracted from the medical records: body weight, body condition score [BCS on a 9-point scale (11)], fecal score (FS, Purina Fecal Score chart, based on a 7-point scale), and the Canine Inflammatory Bowel Disease Activity Index (CIBDAI), including both the total score and individual parameters (activity, appetite, vomiting, weight loss, fecal consistency, and defecation frequency) (12). Dogs met the inclusion criteria for the study if at the time of examination: 1) they exhibited clinical signs consistent with chronic enteropathy (primarily vomiting, diarrhea, or both) for more than 3 weeks; 2) laboratory testing, including complete blood count (CBC), comprehensive serum chemistry profile (including fructosamine and serum total calcium), and urinalysis with urine protein: creatinine ratio, had been performed; 3) they were not receiving any dietary supplements or drugs known to affect calcium metabolism (e.g., calcium carbonate, vitamin D, glucocorticoids), and thus their only calcium intake was derived from their habitual diet. Exclusion criteria included incomplete datasets, as defined by the study protocol and the evidence of concurrent diseases (including protein-losing nephropathy, chronic kidney disease, diabetes, acute pancreatitis, exocrine pancreatic insufficiency, urolithiasis and urinary tract infections). Also considered a reason for exclusion were vague or imprecise information regarding the specific product or at least the category (e.g., commercial diet with hydrolyzed protein, highly digestible diet, novel protein diet), as well as the administration of unbalanced home-prepared diets.

Based on information documented in their medical records during subsequent follow-up after the diagnosis of CE, dogs were grouped into four main categories [according to Dupouy-Manescau et al. (2)]: food-responsive enteropathy (FRE), defined by a positive response to one or more diet trials proposed as the initial therapeutic approach; microbiota-related modulation-responsive enteropathies (MrMRE), referring to dogs that did not respond fully to at least 2 diet trials but showed clinical improvement following treatment with probiotics and/or fecal microbiota transplantation, proposed as adjunct therapies to dietary trials; immunosuppressant-responsive enteropathy (IRE), defined by a positive response to glucocorticoids (prednisolone) and/or another immunosuppressant (cyclosporine), introduced due to a lack of clinical response to dietary and/or microbiota-targeted therapy. A subset of dogs with evidence of hypoproteinemia (albumin < 2.75 g/dl, total protein < 5.60 g/dl), in the absence of proteinuria determined by urinalysis, or other clinicopathological findings indicative of protein loss through non-intestinal routes, such as hepatic dysfunction, was classified as having PLE.

Serum samples from the dogs included in the study were obtained via venous blood collection using a vacuum system. The samples were collected at the time of the clinical evaluation, which was performed to investigate chronic gastrointestinal signs. The veins used for blood sampling included the jugular, cephalic, or saphenous veins. Blood samples were allowed to clot in dedicated serum-separator tubes and then centrifuged at 3,000 × g for 10 min. The resulting serum was immediately transferred into plastic tubes and stored at −20 °C in the clinical laboratory of the Veterinary Teaching Hospital, with storage time before 25(OH)D analysis of up to 24 months. All blood analyses were carried out using standard laboratory methods at the referral institution's medical laboratory; CBC was performed with an automated hematology analyzer (ADVIA 2120, Siemens Healthcare Diagnostics, Tarrytown NY, USA), while chemistry parameters were carried out on an automated chemistry analyzer (AU480, Beckman Coulter/Olympus, Brea, California, USA). Serum fructosamine analysis was performed using a colorimetric nitroblue tetrazolium reduction method (17350H, Sentinel Diagnostics, Milan, Italy).

The quantification of 25(OH)D was performed on stored serum samples using a validated Ultra-High Performance Liquid Chromatography–Tandem Mass Spectrometry (UHPLC–MS/MS) analytical procedure, as reported by Bardhi et al. (13). The method had a lower limit of quantification (LLOQ) of 10 ng/ml. The quantification of 25(OH)D has been previously performed in a population of 40 healthy dogs, which ranged from 13 to 45 ng/ml (13). Healthy dogs, mainly blood donors presented for annual check-up [median body weight 30 kg (range: 3–78 kg); male *n* = 18, female *n* = 22; intact *n* = 19, castrated *n* = 21] were used in this study as a control group.

### Statistical analysis

All data were described using standard descriptive statistics and reported as median and range (minimum and maximum) or mean ± standard deviation for non-normal and normal distributions, respectively. The normality of continuous variables was assessed using the D'Agostino–Pearson test. Age, body weight, BCS, CIBDAI, FS, and serum 25(OH)D were compared among clinical classification groups using ANOVA or Kruskal–Wallis test, depending on data distribution. When significant differences were detected, appropriate *post hoc* analyses (Tukey' or Dunn's test) were performed. Univariate linear regression was initially performed to explore associations between 25(OH)D and the following individual variables: age (months), BCS, FS and CIBDAI scores; hematocrit (Hct), white blood cell count (neutrophils, lymphocytes, monocytes, eosinophils) and platelet count; concentrations of fructosamine, total bilirubin, total protein, albumin, cholesterol, triglycerides, creatinine, urea, total calcium, phosphate, sodium, potassium, chloride, C-reactive protein (CRP), alanine aminotransferase (ALT), and aspartate aminotransferase (AST). Variables showing potential associations (*P* < 0.05) were entered into a multivariable linear regression model using backward selection, with factors removed from the model if *p*-value > 0.20. The coefficient of determination (*R*^2^), adjusted *R*^2^, residual standard deviation, and variance inflation factor (VIF) were used to evaluate model performance and multicollinearity. Standardized regression coefficients (β) were calculated to facilitate graphical comparison of effect sizes, enabling interpretation of the relative influence of each predictor on 25(OH)D. Significance was set at *p* < 0.05. Statistical analyses were performed using GraphPad Prism version 9.2 (GraphPad Software, San Diego, CA, USA) and MedCalc Statistical Software (MedCalc for Windows, version 9.5.0.0, Mariakerke, Belgium).

## Results

A total of 91 dogs met the inclusion criteria, distributed across categories as follows: *n* = 39 (43%) FRE, *n* = 26 (28%) MrMRE, *n* = 16 (18%) IRE, *n* = 10 (11%) PLE. The assessment of medical records allowed for a more detailed characterization of the dogs in each group at the time of inclusion in the study (Table 1).Dogs were classified as FRE following a positive response to one (*n* = 9), two (*n* = 23), or three (*n* = 7) dietary trials. Clinical signs resolved with the following diets: hydrolyzed protein diets (*n* = 17), homemade diets (*n* = 14), gastrointestinal diets (*n* = 4), and novel protein diets (*n* = 4). Within the MrMRE group, 17 dogs showed clinical resolution after treatment with probiotics, while 9 received fecal microbiota transplantation. In the IRE group, 12 dogs were treated with prednisolone alone, and 4 received a combination of prednisolone and cyclosporine. Dogs diagnosed with PLE were managed with a low-fat diet and one of the following treatments: prednisolone (*n* = 7); a combination of prednisolone and cyclosporine (*n* = 2); or a low-fat diet combined with clopidogrel without immunosuppressants (*n* = 1). The majority were purebred (66/91, 72%), with 32 different breeds represented in the overall population (Supplementary material). When classified by category, mixed-breed dogs were the most represented in the FRE, MrMRE, and PLE groups, accounting for 26% (10/39), 31% (8/26), and 30% (3/10) of cases, respectively. In the IRE group, German Shepherds and mixed-breed dogs were equally represented, each comprising 25% (4/16) of the cases. In the overall population (Table 1), 59% (54/91) of the dogs were male (*n* = 39 intact, *n* = 15 neutered). A similar male predominance was observed across all categories, with the exception of the PLE group, where females accounted for 60% (6/10) of the cases (*n* = 5 spayed, *n* = 1 intact).

**Table 1:** Therapeutic interventions applied in 91 dogs with chronic enteropathy grouped according to clinical classification: food-responsive enteropathy (FRE), microbiota-related modulation-responsive enteropathy (MrMRE), immunosuppressant-responsive enteropathy (IRE), and protein-losing enteropathy (PLE).

**FRE (*****n*** = **39)**	
Number of dietary trials required for clinical response	Number of dogs, n (%)
1	9 (23%)
2	23 (59%)
3	7 (18%)
Diet type leading to clinical resolution^a^	Number of dogs, *n* (%)
Hydrolyzed protein diet	17 (44%)
Homemade diet	14 (36%)
Gastrointestinal diet	4 (10%)
Novel protein diet	4 (10%)
**MrMRE (*****n*** = **26)**	
Microbiota-modulating strategy	Number of dogs, *n* (%)
Probiotics	17 (65%)
Fecal microbiota transplantation	9 (35%)
**IRE (*****n*** = **16)**	
Pharmacological treatments used	Number of dogs, *n* (%)
Prednisolone	12 (75%)
Prednisolone + cyclosporine	4 (25%)
**PLE (*****n*** = **10)**	
Pharmacological treatments used in association with a low-fat diet^a^	Number of dogs, n (%)
Prednisolone	7 (70 %)
Prednisolone + cyclosporine	2 (20 %)
Clopidogrel	1 (10 %)

^a^Hydrolyzed protein, gastrointestinal, and low-fat diets refer to commercially available veterinary therapeutic diets; novel protein diets refer to over-the-counter diets based on novel ingredients as sources of protein and carbohydrate. Homemade diets were complete, balanced diets (formulated by a board-certified nutritionist) based primarily on horse or rabbit as the novel protein source, and potato as the main carbohydrate source.

**Figure 1:**
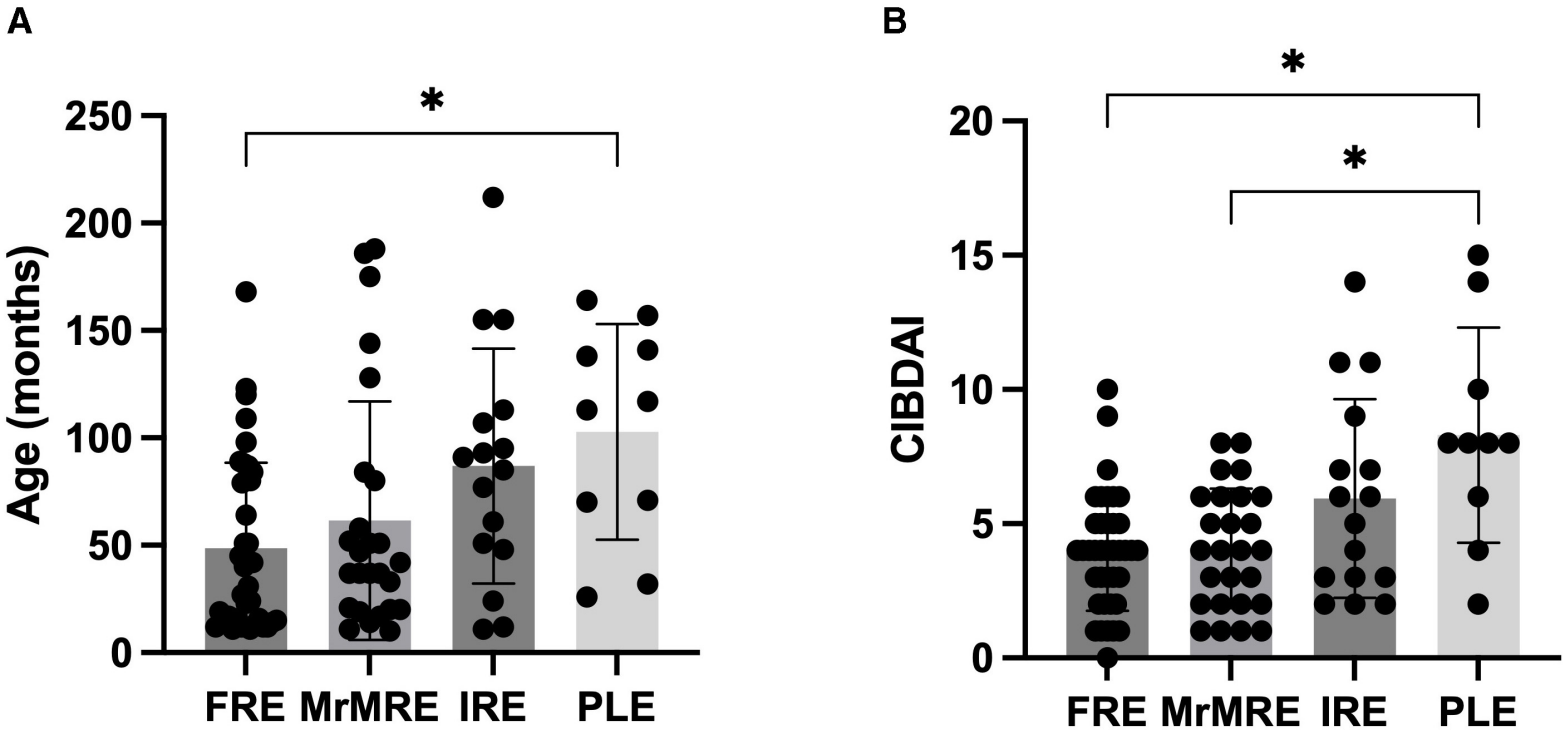
**(A)** Age (in months) and **(B)** CIBDAI score in dogs with chronic enteropathy, grouped according to clinical classification: food-responsive enteropathy (FRE), microbiota-related modulation-responsive enteropathy (MrMRE), immunosuppressant-responsive enteropathy (IRE), and protein-losing enteropathy (PLE). Boxes indicate the interquartile range (25th to 75th percentiles), and whiskers represent the interquartile range. Asterisks denote statistically significant differences, with significance set as *p* < 0.05.

Data on age (months), body weight (kg), BCS, CIBDAI, and FS were compared among the different categories (Table 2). A significant difference in age was observed among groups (*p* = 0.006), with dogs in the FRE group being younger than those in the PLE group [median 40 months [11–168] vs. 115 months [26–164] months; *p* = 0.019]. No significant differences in age were found between FRE and the other groups (Figure 1A). CIBDAI scores also differed significantly among groups (*p* = 0.003), with PLE dogs showing higher values [median 8 [2–15]] compared to FRE [median 4 (0–10); *p* = 0.04] and MrMRE [median 4 [1–8]; *p* = 0.049; Figure 1B]. No significant differences were found for body weight, BCS, or FS (Table 1).

**Table 2:** Descriptive statistic and comparison of age, sex, and clinical scores in 91 dogs with chronic enteropathy grouped according to clinical classification: food-responsive enteropathy (FRE), microbiota-related modulation-responsive enteropathy (MrMRE), immunosuppressant-responsive enteropathy (IRE), and protein-losing enteropathy (PLE).

**Clinical data**	**FRE**	**MrMRE**	**IRE**	**PLE**	***p*-value**
Sex (*n*)	*m* = 20; *c* = 5 *f* = 9; *s* = 5	*m* = 9; *c* = 7 *f* = 4; *s* = 6	*m* = 8; *c* = 1 *f* = 1; *s* = 6	*m* = 2; *c* = 2 *f* = 1; *s* = 5	-
Age (months)	40 (11-168)^a^	39 (11–188)^ab^	88 (11–212)^ab^	115 (26-164)^b^	0.006
Body weight (kg)	14 (3.5–43)	14 (3–37)	13 (1.6–31)	12 (5.4–38)	0.727
Body condition score	4 (2–6)	4 (3–7)	4 (1–5)	3 (2–9)	0.119
CIBDAI	4 (0–10)^a^	4 (1–8)^a^	5 (2–14)^ab^	8 (2–15)^b^	0.003
Fecal score	4 (2–6)	5 (2–7)	3 (2–7)	6 (4–7)	0.101

The data show the median and range.

m = male; C = castrated male; f = female; s = spayed female. Different letters indicate statistically significant differences (*p* < 0.05).

**Figure 2:**
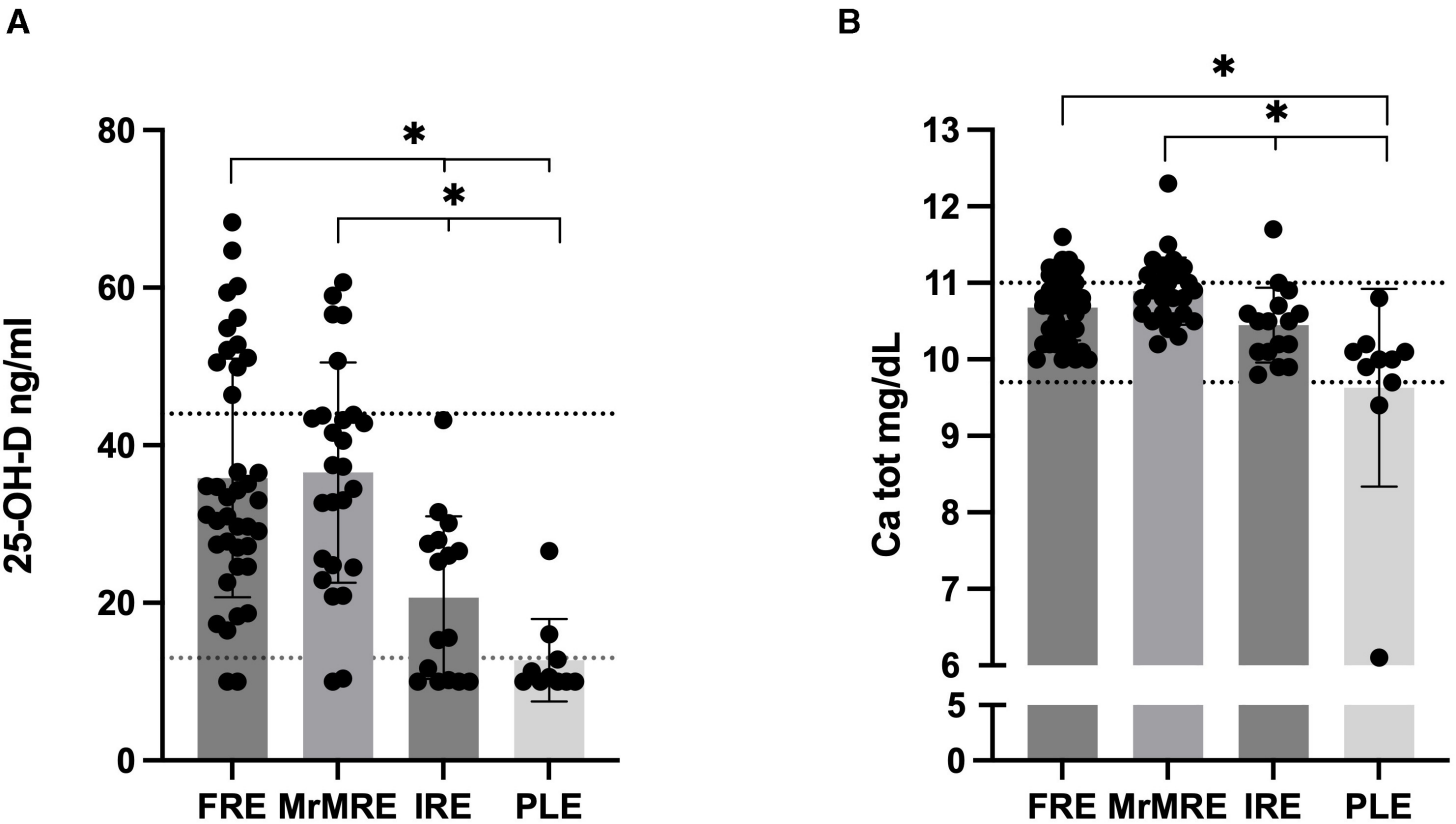
Serum **(A)** 25(OH)D and **(B)** total calcium concentration in dogs with chronic enteropathy, grouped according to clinical classification: food-responsive enteropathy (FRE), microbiota-related modulation-responsive enteropathy (MrMRE), immunosuppressant-responsive enteropathy (IRE), and protein-losing enteropathy (PLE). Boxes indicate the interquartile range (25th to 75th percentiles), and whiskers represent the interquartile range. Asterisks denote statistically significant differences, with significance set as p < 0.05. The dashed line indicates **(A)** the reference interval derived from a population of 40 healthy dogs (13), **(B)** the established reference range for total calcium (9.7–11 mg/dL).

Serum 25(OH)D concentrations differed significantly among groups (*p* < 0.001; Figure 2A). Values were significantly lower in the PLE group [median 10.3 [10–27]] compared to FRE [median 33 [10–68], *p* < 0.001] and MrMRE [median 37 [10–61], *p* < 0.001]. Similarly, dogs in the IRE group (median 20 [10–43]) showed lower concentrations when compared with FRE and MrMRE (*p* = 0.006 for both). No significant differences were found between FRE and MrMRE, or between IRE and PLE.

Serum 25(OH)D concentrations in CE dogs were compared with a control group of 40 healthy dogs from a previous study (4). From this analysis, 31% of FRE and 19% of MrMRE dogs had 25(OH)D concentrations exceeding those of healthy controls. However, no statistically significant differences were observed between healthy, FRE, and MrMRE groups (data not shown). In contrast, none of the IRE or PLE dogs exhibited values above the established reference range (13–45 ng/mL). Significant variation in total serum calcium levels among groups were identified (*p* < 0.001; Figure 2B). Consistent with 25(OH)D data, in the PLE group dogs had lower total calcium level [median 10 (6.1–10.8) mg/dL] compared to FRE [median 10.7 (10–11.6) mg/dL, *p* = 0.001] and MrMRE [median 10.9 (10.2–12.3) mg/dL, *p* < 0.0001]. Relative to the MrMRE group, dogs in the IRE group were likewise found to have lower calcium concentrations [median 10.5 (9.80–11.7) mg/dL, *p* = 0.022]. Only three dogs had a serum total calcium level below or equal to the low reference range. All of these dogs belonged to the PLE group.

**Figure 3:**
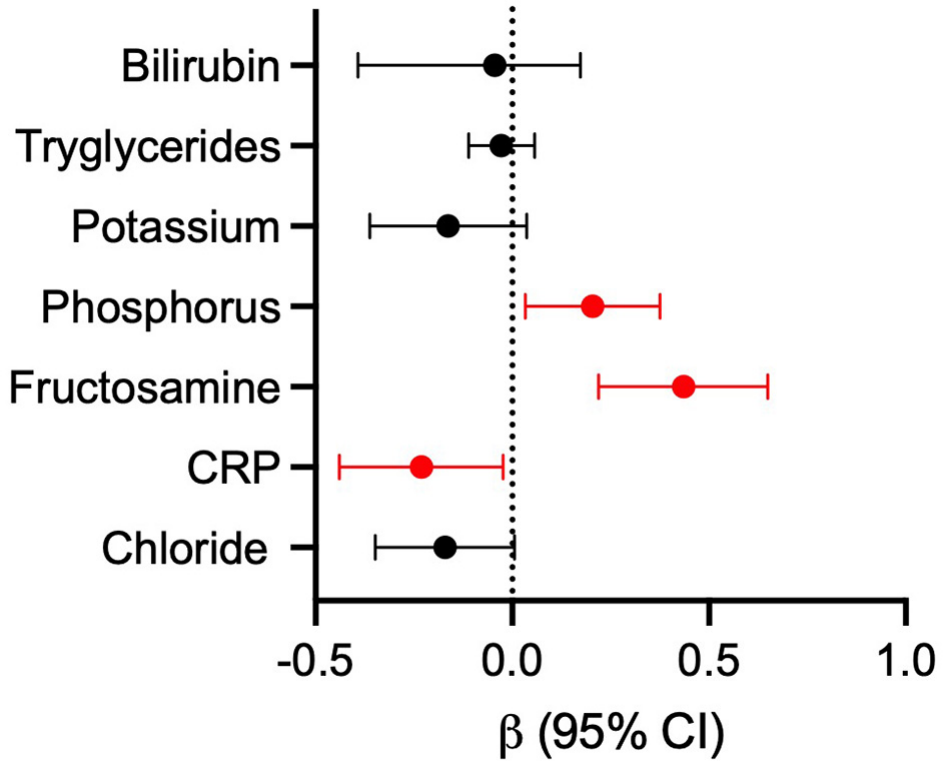
Forest plot of standardized β coefficients with 95% CI calculated from the linear regression model. Effect sizes represent the relative influence of each variable on 25(OH)D. Significant values are highlighted (*p* < 0.05). CRP, C-reactive protein.

A backward multiple linear regression was performed to identify factors associated with serum 25(OH)D concentrations in 75 dogs. Indeed, of the 91 dogs included in the study, 75 had a complete dataset with all the variables required for the analysis. The following parameters were initially included but were excluded during backward elimination due to a *p*-value > 0.20: FS, BCS, hematological parameters, ALT, AST, albumin, total protein, sodium, cholesterol, creatinine, urea and total calcium. CRP, phosphorus, and fructosamine were identified as independent predictors of 25(OH)D concentration, while chloride, triglycerides, total bilirubin, and potassium were retained in the final model as covariates without a statistically significant independent effect (*R*^2^ = 0.418; adjusted *R*^2^ = 0.357; *p* < 0.0001, Table 3). Figure 3 illustrates the effect size of each variable included in the model, expressed as standardized β coefficients with 95% confidence intervals. Lower serum fructosamine (β = 0.435, CI: 0.219 to 0.650, *p* < 0.001) and phosphorus (β = 0.204, CI: 0.033 to 0.376, *p* < 0.020) were associated with a lower 25(OH)D, whereas higher CRP (β = −0.231, CI: −0.440 to −0.023, *p* < 0.030) was associated with a lower 25(OH)D. Abnormal serum fructosamine concentrations were found in 14% of the dogs. Of these, 12/91 had fructosamine concentrations below the reference range (Table 3). Eleven out of the 12 with low fructosamine concentrations were in the PLE group and one was in the IRE group (with low albumin and normal total protein).

**Table 3:** Median (range) and multivariable linear regression model of seven factors associated to serum 25(OH)D concentration.

**Dogs, *n* = 75 *R*^2^ = 0.42**	**Median (range)**	**Coefficient**	**Standard error**	**95% CI**		***p*-value**
				**Lower**	**Upper**	
Chloride [RI: 108–118 mEq/L]	114 (103–128)	−0.80	0.413	−1.624	0.024	0.057
C-reactive protein [RI: 0–0.85 mg/dl]	1.06 0.61–29	−0.738	0.332	−1.40	−0.075	0.030^*^
Fructosamine [RI: of 222–382 μmol/l]	296 (110–421)	0.105	0.026	0.053	0.157	< 0.001^*^
Phosphorus [RI: 2.65–5.40 mg/dl]	3.97 (1.91–8.55)	3.336	1.397	0.541	6.132	0.020^*^
Potassium [RI: 3.8–5.0 mEq/l]	4.4 (2.7–6.5)	−5.005	3.063	−11.114	1.104	0.107
Triglycerides [RI: 30–120 mg/dl]	55 (29–388)	−0.038	0.029	−0.096	0.019	0.195
Total bilirubin [RI: 0.07–0.33 mg/dl]	0.19 (0.03–0.61)	36.190	22.734	−9.152	81.533	0.116

^*^p < 0.05; RI, reference interval.

## Discussion

Hypovitaminosis D is commonly observed in dogs with CE (4–8). In this study, serum 25(OH)D corresponded with the classification of dogs with CE based on clinical condition and therapeutic response, with lower concentrations observed in dogs exhibiting more severe CE phenotypes. In dogs with PLE, low serum 25(OH)D concentration has been identified as a negative prognostic factor (3, 9), and dogs that died or were euthanized had lower vitamin D concentrations compared to those that survived (9). Our study cannot be considered a prognostic investigation, as the evaluation of medical records was not designed to assess potential associations with mortality. However, dogs in the IRE and PLE groups, typically associated with more severe clinical scores and a generally poorer prognosis (3, 10, 14), showed the lowest vitamin D concentrations, in contrast to dogs in the FRE and MrMRE groups, which had better clinical scores. Although the association between hypovitaminosis D and CE in dogs is well recognized, evidence supporting clinical improvement due to vitamin D supplementation in cases with confirmed deficiency remains currently lacking (15, 16). Interestingly, a rapid increase in 25(OH)D levels has been observed in PLE dogs that were not treated with cholecalciferol supplementation but rather received standard treatment for their underlying condition (15). This suggests that clinical improvement itself may play a role in raising 25(OH)D levels. Moreover, a major difficulty in defining a supplementation protocol is that an exact cut-off value for deficiency has not yet been defined for dogs (10, 16). In humans, instead, increasing evidence suggests that an optimal threshold lies between 30 and 40 ng/ml (17, 18), while lower levels are linked to increased risk of cardiovascular, immune, metabolic, and neoplastic diseases, as well as higher all-cause mortality (19). Although there is currently no evidence that vitamin D supplementation improves the clinical condition of dogs with CE and PLE (15, 16), it has been shown to effectively increase serum 25(OH)D concentrations in deficient dogs within the first month of feeding a vitamin D3-enriched diet (20). In our study, serum 25(OH)D concentrations showed a greater variability both within CE groups and among healthy dogs, with a relatively high proportion of FRE and MrMRE dogs with values exceeding those of healthy dogs, although this difference was not statistically significant. Even though our study population may not be fully representative of a broader CE population, it covered a variety of dog breeds, ranging in size from very small to large, similarly to previous studies (4, 7). Instead, the majority of the healthy dog population consisted of medium-to-large breeds, which could limit the generalizability of the results. Consistent with previous studies evaluating vitamin D status in healthy dogs (21, 22), and in dogs with disease (4, 7, 23), age was not predictive of serum 25(OH)D concentration in our study. In dogs, serum vitamin D concentrations depend heavily on dietary intake, and available data suggest that serum 25(OH)D concentrations in dogs vary markedly according to diet brand and supplementation used (24). Although dogs receiving vitamin D–containing supplements were excluded from the study, a complete record of the specific diets fed at the time of serum sampling was not available for all dogs. While direct comparisons between the vitamin D content of GI therapeutic diets and standard maintenance diets are currently lacking, it is plausible that the former might contain higher vitamin D concentrations than standard maintenance diets, given that veterinary GI diets are formulated to address malabsorption and support mucosal recovery.

Vitamin D plays a key role in mineral homeostasis, primarily by enhancing intestinal absorption of calcium and phosphorus. Higher serum 25(OH)D concentrations are usually linked to greater availability of the active form of vitamin D (1,25(OH)2D), which promotes increased intestinal uptake of both minerals by the intestine (25, 26). This physiological mechanism may explain the positive association observed between serum 25(OH)D and phosphorus concentrations in our study. Despite calcium's central role in vitamin D metabolism, total serum calcium was excluded from the final regression model. Consequently, the lack of association between total calcium and vitamin D status may be due to the limitations of using total calcium as the sole marker of calcemia in this study. This is because total calcium does not accurately represent the biologically active fraction of calcium, and it is influenced by serum albumin and other protein-bound components. Total serum calcium is often decreased in CE dogs (4, 6, 27), but it is not a specific or standalone marker for diagnosis or prognosis. In this study population, however, total calcium levels reflected the clinical characteristics of CE dogs, with lower levels observed in those with IRE and PLE. That being said, ionized calcium (iCa), which more reliably reflects the physiologically active form (26), would have been a more appropriate parameter for assessing calcium status in this context.

Parathyroid hormone (PTH) measurement plays a key role in assessing vitamin D status, as elevated PTH concentrations can indicate a deficiency in the active form of vitamin D, even when serum vitamin D concentrations appear borderline or low-normal (25, 26). As iCa and PTH measurements were not available for the dogs in this study, we are unable to fully evaluate their vitamin D and calcium status.

In general terms, it's unclear whether low vitamin D contributes to chronic inflammatory enteropathy development or is merely a consequence of it. Emerging evidence suggests that hypovitaminosis D may also play a causative role in the onset of factors involved in the multifactorial etiology of CE, such as intestinal inflammation and immune system dysregulation (28–30). Low vitamin D status has been negatively associated with systemic and gastrointestinal inflammation in dogs with CE (7). Elevated inflammatory markers, including neutrophil and monocyte counts, as well as pro-inflammatory cytokines, such as IL-2 and IL-8, have been associated with low serum 25(OH)D concentrations, reinforcing the link between inflammation and vitamin D deficiency (7).

CRP is an acute phase protein, that rises in response to systemic inflammation (31), and can be measured as part of the diagnostic workup in dogs with CE. When measured within 1–3 days of hospitalization in PLE dogs, CRP was identified as a negative prognostic factor, with higher concentrations being observed in dogs with a higher mortality rate (32). In our study, low vitamin D concentrations were associated with higher CRP values, and a similar inverse relationship between vitamin D status has been observed in dogs presenting with a hemoabdomen (33).

Albumin is a negative acute phase protein and a negative prognostic factor in dogs with CE (3, 31). A low albumin concentration has been directly linked to vitamin D deficiency (4, 15), and dogs with hypoalbuminemia, such as those with PLE, might be at an increased risk of deficiency depending on the severity of their condition (5, 15, 34). Intestinal malabsorption or loss of vitamin D-binding protein has been proposed as a possible cause of hypovitaminosis D in dogs with inflammatory bowel disease and hypoalbuminemia (5, 8). Contrary to the findings of a recent study on PLE dogs (15), in this study albumin concentrations were not associated with vitamin D status in the overall population, while vitamin D concentrations were reduced in both PLE and IRE dogs compared to FRE and MrMRE groups. Consistent with our findings, a study investigating hypovitaminosis D in dogs with Spirocercosis reported low vitamin D concentrations in all affected dogs, regardless of albumin concentrations (23). Compared to healthy controls, decreased albumin concentration was observed only in dogs exhibiting the neoplastic form and presenting with diarrhea (23).

In this study, the positive association between serum 25(OH)D and fructosamine concentrations may be affected by the status of serum protein, particularly albumin. While fructosamine is primarily used to monitor blood glucose in diabetic animals, its concentration is also influenced by protein concentrations (35). Vitamin D is partly bound to albumin for transport in the bloodstream, while fructosamine reflects the glycation of serum proteins, primarily albumin. In dogs with low protein concentrations, serum fructosamine tends to be lower than normal, and the degree of this decrease can provide some insight into the duration of the protein deficiency (35, 36). In this population of CE dogs, hypoalbuminemia was a feature of PLE dogs, and the correlation between vitamin D and fructosamine may largely reflect variations in protein availability and poorer protein status, rather than glycemic control *per se*.

Although fructosamine and CRP were the most significant predictors, other variables, such as chloride, triglycerides, total bilirubin and potassium, were retained in the final regression model. While these variables did not demonstrate independent statistical significance, their inclusion may reflect underlying physiological interactions, homeostatic mechanisms or correlations with the primary predictors. This pattern highlights the multifactorial regulation of 25(OH)D concentrations and indicates that various metabolic and inflammatory factors may influence vitamin D status collectively.

The retrospective design of this study led to several limitations. Firstly, there was a lack of standardization in pharmacological and dietary treatments administered to the dogs, as well as in the laboratory parameters available in the medical records. In addition, the inclusion of only cases with complete datasets may have introduced a potential selection bias, arising both from the accuracy and completeness of medical record compilation and from the precision of the information provided by the owners, which could ultimately limit the representativeness of the general population. Lastly, there was a lack of iCa and PTH measurements, which prevented a more complete evaluation of vitamin D and calcium status in the study population.

## Conclusion

This retrospective study provides valuable insights into the vitamin D status of dogs with chronic enteropathies, showing differences in vitamin D levels among dogs which responded to different treatment options. Notably, CRP and fructosamine, which are easily available parameters, might represent potential correlates of vitamin D concentration and warrant further validation. Further research is needed to clarify the long-term effects of vitamin D deficiency in dogs with chronic enteropathies, and to establish whether vitamin D supplementation could be beneficial.

## Data Availability

The raw data supporting the conclusions of this article will be made available by the authors, without undue reservation.
