# Supplementary material for: Serum 25(OH)D reflects clinical characterization in dogs with chronic enteropathies
Source: Front Vet Sci. 2025 Oct 17;12:1677939. doi: 10.3389/fvets.2025.1677939 (PMC12575127; doi:10.3389/fvets.2025.1677939)
Supplement: Supplementary file 1 [file Table_1.docx]

Supplementary Material

# Supplementary Data

Breed distribution of dogs included in the study (n=91), listed in descending order of frequency.

| Breed | Number of dogs per each breed |
| --- | --- |
| Maltese | 7 |
| German Shepherd, Poodle | 5 |
| American Bully, Border Collie, Cavalier King Charles Spaniel, Cocker Spaniel, English Setter, French Bulldog, Labrador Retriever, Australian Shepherd | 3 |
| Boxer, Dachshund, Golden Retriever | 2 |
| Beagle, Belgian Shepherd, Bernese Mountain Dog, Bolognese, Cairn Terrier, English Pointer, Italian Hound, Jack Russell Terrier, Papillon, Parson Russell Terrier, Pinscher, Pug, Samoyed, Shih Tzu, Springer Spaniel, Welsh Corgi, Weimaraner, West Highland White Terrier, and Yorkshire Terrier | 1 |
| Mixed-breed | 25 |
